# Comparison of Autonomic Reactions during Urodynamic Examination in Patients with Spinal Cord Injuries and Able-Bodied Subjects

**DOI:** 10.1371/journal.pone.0161976

**Published:** 2016-08-30

**Authors:** Yu-Hui Huang, Hsiao-Yun Chang, Sen-Wei Tsai, Li-Wei Chou, Sung-Lang Chen, Yu-Hao Lin

**Affiliations:** 1 Department of Physical Medicine & Rehabilitation, Chung Shan Medical University Hospital, Taichung, Taiwan; 2 School of Medicine, Chung Shan Medical University, Taichung, Taiwan; 3 School of Physical Therapy, Chung Shan Medical University, Taichung, Taiwan; 4 Room of Physical Therapy, Chung Shan Medical University Hospital, Taichung, Taiwan; 5 Department of Physical Medicine and Rehabilitation, Taichung Tzu Chi Hospital, Buddhist Tzu Chi Medical Foundation, Taichung, Taiwan; 6 Department of Physical Medicine and Rehabilitation, School of Medicine, Tzu Chi University, Hualien, Taiwan; 7 Department of Physical Medicine and Rehabilitation, China Medical University Hospital, Taichung, Taiwan; 8 School of Chinese Medicine, College of Chinese Medicine, China Medical University, Taichung, Taiwan; 9 Department of Urology, Chung Shan Medical University Hospital, Taichung, Taiwan; University of Missouri Columbia, UNITED STATES

## Abstract

**Background/Purpose:**

This study compares heart rate variability (HRV) and systolic blood pressure (SBP) changes of spinal cord injury (SCI) patients during urodynamic study (UDS) with able-bodied controls.

**Methods:**

Twenty four complete suprasacral SCI patients (12 tetraplegia and 12 paraplegia) and 12 age-matched able-bodied volunteers received BP and HRV evaluation throughout urodynamic examination. We chose seven time points during the examinations: resting, Foley catheter insertion, start of infusion, and infused volume reaching 1/4, 2/4, 3/4 and 4/4 of maximal capacity. At each time point, electrocardiogram with a duration of 5 min was used for power spectral density analysis of HRV.

**Results:**

Only control subjects displayed significant elevation of SBP during Foley catheter insertion compared to resting values. Both control and tetraplegic groups experienced significant elevation of SBP at maximal bladder capacity compared to resting values. Tetraplegic values were also significantly greater than the other two groups. Control subjects displayed significant elevation of low frequency/high frequency (LF/HF) ratios during Foley catheter insertion and when approaching maximum bladder capacity. These findings were not seen in the paraplegic and tetraplegic groups. However, subgroup analysis of tetraplegic subjects with SBP elevation >50 mmHg demonstrated a similar LF/HF response to the able-bodied controls.

**Conclusion:**

Tetraplegic patients experienced BP elevation but did not experience significant changes in HRV during bladder distension. This finding may imply that different neurological pathways contribute to AD reaction and HRV changes during bladder distension. However, profound AD during UDS in tetraplegic patients was associated with corresponding changes in HRV. Whether HRV monitoring would be beneficial in SCI patients presenting with significant AD, it needs further studies to elucidate.

## Introduction

Autonomic dysfunction frequently accompanies spinal cord injuries (SCI). Strong correlations between the level of injury and the severity of autonomic dysfunction have been reported[1]. SCI patients may also experience autonomic dysreflexia (AD), which causes autonomic dysfunction. AD is commonly defined as an abrupt 20 to 40 mmHg rise in systolic blood pressure (SBP) above the baseline blood pressure with or without other symptoms[2]. Almost all SCI patients suffer from neurogenic lower urinary tract dysfunction (NLUTD) and therefore urodynamic study (UDS) is essential for these patients in order to assess the bladder function during their initial rehabilitation period and the following lifelong surveillance[3]. European Association of Urology guidelines on NLUTD also demonstrate grade A recommendation for the necessity of UDS to document the dysfunction of lower urinary tract[4]. Several studies, including our previous report, have documented the presence of AD with noticeable SBP increase during UDS in SCI patients [5–7].

Heart rate variability (HRV) was developed as a non-invasive, practical and reproducible method for quantitative evaluation of autonomic activity[8]. HRV measures the spontaneous change of the R-R interval in continuous electrocardiogram (ECG) as a response to physiologic demand. It was initially used to evaluate autonomic function in cardiovascular diseases[9]. Vagal stimulation allows for beat-to-beat control of the heart rate, whereas sympathetic stimulation has a more gradual effect on the heart rate[10]. The frequency domain analysis has been used to subdivide the variability of HR into different frequency components and to quantify the variance or “power” at each specific frequency.[11] Power spectral density analysis of HRV yields a high frequency (HF, 0.15~0.4 Hz) component reflecting vagal modulation and a low frequency (LF, 0.04~0.15Hz) component that is influenced by both sympathetic and vagal outflows, while the ratio of the two frequencies (LF/HF) is thought to assess sympatho-vagal balance [12,13].

Research has assessed the autonomic dysfunction in traumatic tetraplegic and paraplegic patients by spectral analysis of HRV and demonstrated it may be used as reproducible indices of autonomic regulation in SCI patients [14,15]. Mehnert et al. was the first and only study to use HRV as an objective measurement to evaluate autonomic function during UDS exclusively in able-bodied subjects[16].

Blood pressure elevation, a sign presenting the occurrence of AD in SCI patients, is usually measured by a sphygmomanometer with an inflatable cuff wrapped around forearm, which is not convenient for continuous monitoring. Although heart rate changes during AD are still debatable in previous studies[6,17,18]. HRV in SCI patients with AD during UDS have yet to be reported. The current study was designed to compare BP and HRV changes during UDS among tetraplegic, paraplegic patients and able-bodied controls; and to evaluate the role of HRV in measuring AD reactions, besides traditional BP measurement, and to explore the possible underlying HRV mechanism during AD events.

## Materials and Methods

We conducted this prospective case-control study in a tertiary hospital affiliated with a medical university from January 2012 to December 2012. The study evaluated SCI patients referred for urodynamic examinations. The inclusion criteria were: 18 to 65 years of age, single and complete cord lesion at cervical (tetraplegia) or thoracic levels (paraplegia), and stable neurologic condition after spinal shock period (judged by increased muscle tone and presence of detrusor contraction or sphincter spasticity). The exclusion criteria were any previous genito-urinary diseases or operations, current symptomatic urinary tract infection (UTI), history of cardio-vascular disease, currently taking cardio-vascular medications (including cardiac glycosides, cholinomimetic, or sympathomimetic drugs), and multiple level spinal cord injuries. A control group of able-bodied volunteers were enrolled with the following criteria: of similar age to the SCI participants and without chronic diseases, neurological diseases or voiding problems. This study was approved by the Chung Shan Medical University Hospital Institutional Review Board (CS11184), and all patients provided written informed consent prior to participation.

All of our participants (able-bodied volunteers and SCI patients) received oral 500mg levofloxacin daily as prophylaxis for 2 days before UDS. The neurological examinations and classification of neurologic injury level and the completeness of injury was based upon criteria from the International standards for neurological classification of spinal cord injury[19]. The execution and reading of UDS used the standards of the International Continence Society[20]. Cystourethrometry was performed by a triple lumen catheter with continuous filling of isotonic saline at a rate of 30 ml per minute at a room temperature of 24 to 26°C. Electromyography of the external urethral sphincter was obtained via surface electrodes placed beside the anus. Filling was stopped if one of the following conditions presented: (1) the patient reported a sensation of fullness, (2) spontaneous urine leakage, (3) the infused volume reached 500 ml, (4) blood pressure reached a dangerous level (180 mmHg systolic or 110 mmHg diastolic blood pressure), and (5) intolerable AD symptoms.

We informed subjects to abstain from caffeine, alcohol, and smoking for 12 hours before testing. Before conducting UDS, we told patients to lie down quietly and relax for 20 minutes. Blood pressure (BP), pulse rate (PR) and 7-lead ECG were recorded throughout the process during the relaxation period and UDS procedures. The BP and PR measurements were obtained from an automatic sphygmomanometer with an inflatable cuff wrapped around the patient’s upper arms and recorded every two minutes. An elevation of SBP for more than 20 mmHg was defined as an AD reaction.[2,6] HRV analysis was performed using Medilog^TM^ Darwin software which can automatically transform the ECG information into power spectrum of different frequency domain. The software subdivided the variability of HR into different frequency components and to quantify the “power” at each specific frequency. Since LF/HF ratio showed good reproducibility and was suggested as the best measure of autonomic nerve activity to the heart, we present only this parameters of HRV[14,21]. We chose seven time points during the examinations, and five-minute ECG intervals around each point (2.5 min before and after the chosen time point) were analyzed for HRV power spectrum analysis[12]. These seven time points were: after lying quietly for 20 min (p1), at Foley catheter insertion (p2), the start of infusion (p3), and at an infused volume of 1/4 (p4), 2/4 (p5), 3/4 (p6) and 4/4 (p7) of the maximum capacity.

We analyzed the differences between the SBP, PR and HRV parameters of the same group at each time point using repeated measures ANOVA (RM-ANOVA). The differences among the groups were analyzed by ANOVA and post-hoc analysis with Tukey's method for continuous variables, and chi-square analysis for category variables. A *p*<0.05 was considered statistically significant.

## Results

Since eligible SCI patients with good compliance to our study protocol were relatively rare, we did our best to enroll all possible cases. A total of 36 subjects were recruited, including 12 SCI patients with complete tetraplegia, 12 SCI patients with complete paraplegia and 12 able-bodied subjects (the control group). Their basic demographics are listed in Table 1. There was no difference in age and gender distribution among groups, and no difference in injury duration between tetraplegic and paraplegic groups. The injury level distributions are 2 in C4, 2 in C5, 5 in C6, 3 in C7 among tetraplegic patients and 1 in T5, 2 in T6, 3 in T7, 4 in T8, 2 in T10 among paraplegic patients.

**Table 1:** Basic demographic data of our subjects.

	Control	Paraplegia	Tetraplegia	p value
Total numbers	12	12	12	
Age (years)	37±8	35±13	36±12	0.923
Gender				
Male	8	8	9	0.856
Female	4	4	3	
Injury duration (months)		27±25	29±28	0.414

Values are given as mean ± standard deviation.

**Fig 1:**
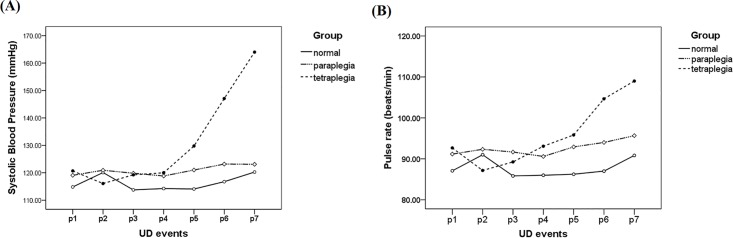
**The changes of SBP (1A) and PR (1B) throughout the examination of these three groups.** These seven time points were: after lying quietly for 15 min (p1), Foley catheter insertion (p2), start of infusion (p3), and infused volume at 1/4 (p4), 2/4 (p5), 3/4 (p6) and 4/4 (p7) of maximum capacity.

The changes of SBP and PR throughout the examination of these three groups are summarized in Table 2 and showed in Fig 1. Compared to the baseline, Foley catheter insertion stimulation (p2) induced statistically significant elevation of SBP and PR in the control group (statistical powers were 0.81 and 0.74) but not in SCI groups. Both the control and tetraplegic groups showed statistically significant elevation in SBP and PR at bladder fullness (p7) as compared to resting values (statistical powers were 0.86 and 0.99). The tetraplegic group experienced more significant SBP and PR elevation than the other two groups during near maximal bladder capacity (p6 and p7) (statistical powers were 0.94 to 0.99). AD, defined as SBP elevation of more than 20 mmHg, was found in all of the tetraplegic patients, one in the paraplegic group, and none in the control group.

**Table 2:** Blood pressure and pulse rate changes at rest, Foley insertion and during the process of bladder infusion.

	Resting (P1)	Foley insertion (P2)	Begin infusion (P3)	1/4 capacity (P4)	2/4 capacity (p5)	3/4 capacity (p6)	Maximal capacity (p7)	F value (RM-ANOVA)	p value
SBP (mmHg)									
Normal	115±13^e^	120±13^e^	114±12	114±12	114±13	117±13^a^	120±11^a^^,^^e^	4.109	0.001**
Paraplegia	119±16	121±19	120±19	119±18	121±19	123±22	123±19^b^	1.674	0.141
Tetraplegia	121±14^f^	116±18	119±9	120±11	130±14	147±29^a^^,^^f^	164±25^a^^,^^b^^,^^f^	33.118	<0.001**
F value (ANOVA)	0.614	0.468	0.807	0.653	2.100	8.234	26.985		
p value	0.547	0.631	0.455	0.527	0.094	0.002*	<0.001*		
PR (beats/min)									
Normal	87±11^g^	91±7^g^	86±8	86±7	86±9	87±8^c^	91±12^c^^,^^g^	6.188	<0.001**
Paraplegia	91±13	92±17	92±16	91±17	93±18	94±18	96±18^d^	2.058	0.101
Tetraplegia	93±12^h^	87±9	89±8	94±11	97±9	107±26^c^	114±29^c^^,^^d^^,^^h^	12.376	<0.001**
F value (ANOVA)	0.925	0.770	0.996	1.292	2.228	6.152	6.708		
p value	0.407	0.471	0.380	0.288	0.117	0.005*	0.004*		

* Statistical significance of ANOVA analysis.

** Statistical significance of RM-ANOVA analysis

^a^ p<0.05, ANOVA test with post hoc test by Tukey’s method, SBP values at p6 and p7 were significantly different between normal and tetraplegia groups (statistical powers are 0.94 and 0.99)

^b^ p<0.05, ANOVA test with post hoc test by Tukey’s method, SBP value at p7 was significantly different between paraplegia and tetraplegia groups (statistical power is 0.99)

^c^ p<0.05, ANOVA test with post hoc test by Tukey’s method, PR values at p6 and p7 were significantly different between normal and tetraplegia groups (statistical powers are 0.79 and 0.81)

^d^ p<0.05, ANOVA test with post hoc test by Tukey’s method, PR value at p7 was significantly different between paraplegia and tetraplegia groups (statistical power is 0.65)

^e^ p<0.05, repeat measurement ANOVA test, SBP values of p2 and p7 were significantly different from baseline (p1) in the normal group (statistical powers are 0.82 and 0.86)

^f^ p<0.05, repeat measurement ANOVA test, SBP values of p6 and p7 were significantly different from baseline (p1) in the tetraplegia group (statistical powers are 0.89 and 0.99)

^g^ p<0.05, repeat measurement ANOVA test, PR values of p2 and p7 were significantly different from baseline (p1) in the normal group (statistical powers are 0.74 and 0.82)

^h^ p<0.05, repeat measurement ANOVA test, PR value of p7 was significantly different from baseline (p1) in the tetraplegia group (statistical power is 0.94)

**Fig 2:**
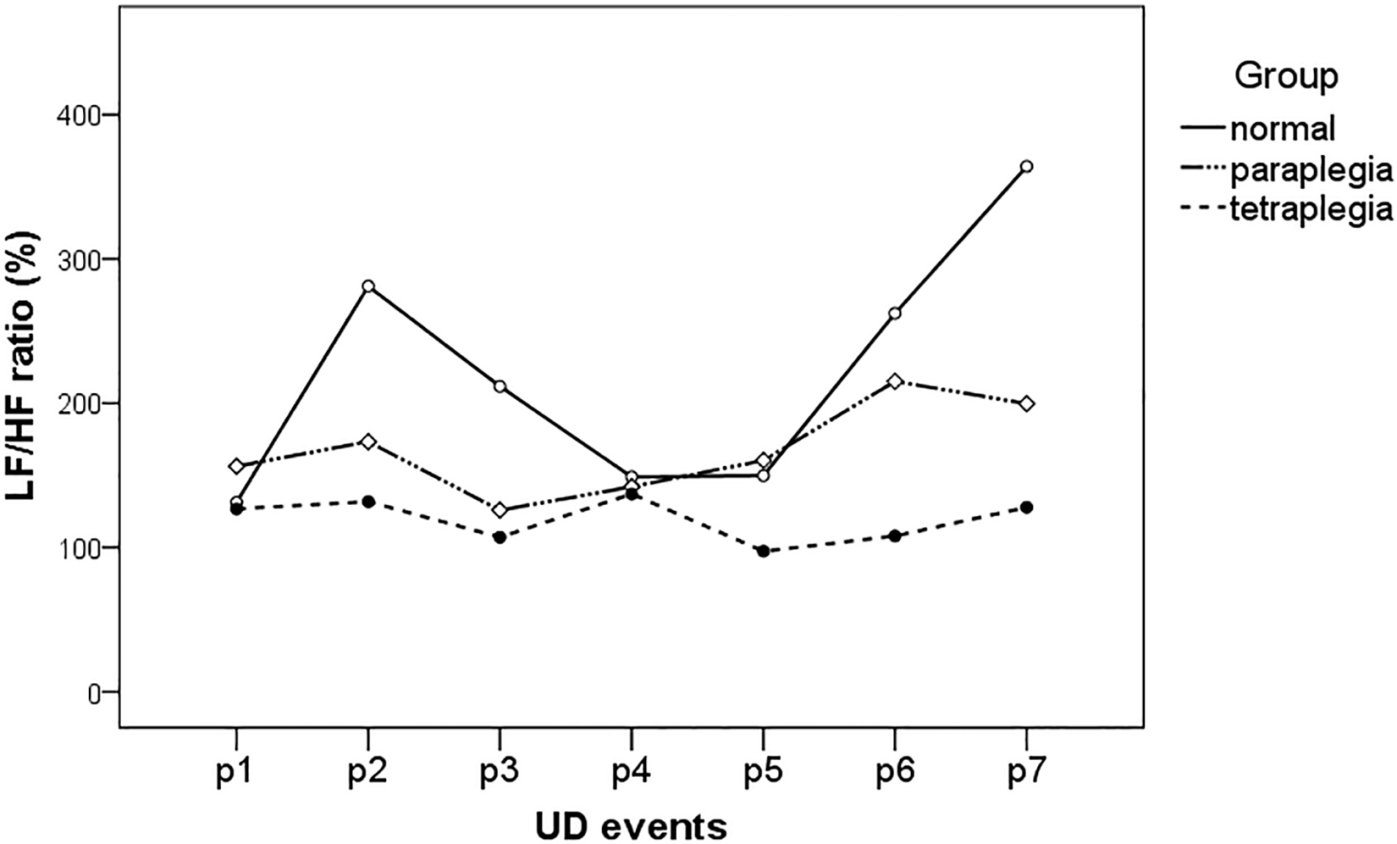
The changes of HRV parameters, LF/HF ratio during UDS of the three groups. These seven time points were: after lying quietly for 15 min (p1), Foley catheter insertion (p2), start of infusion (p3), and infused volume at 1/4 (p4), 2/4 (p5), 3/4 (p6) and 4/4 (p7) of maximum capacity.

Table 3 lists the HRV parameters, LF/HF ratio, during UDS of the three groups, which are also illustrated in Fig 2. The control group experienced significant elevated LF/HF ratio during Foley catheter insertion (p2) and at near maximal bladder capacity (p6 and p7) as compared to baseline (p1) (statistical powers were 0.95 to 0.99). These findings were not seen in SCI patients, neither the paraplegic nor the tetraplegic group. There were significant differences between the control and SCI groups in LF/HF ratio at p2, p6 and p7 (statistical powers were 0.69 to 0.72 for paraplegia versus normal and 0.91 to 0.99 for tetraplegia versus normal).

**Table 3:** Power spectrum analysis (LF/HF ratio) of HRV at rest, Foley insertion and during the process of bladder infusion.

	Resting (P1)	Foley insertion (P2)	Begin infusion (P3)	1/4 capacity (P4)	2/4 capacity (p5)	3/4 capacity (p6)	Maximal capacity (p7)	F value (RM-ANOVA)	p value
Normal	131±73^c^	298±124^a^^,^^b^^,^^c^	245±191	149±36	150±27	262±61^a^^,^^c^	364±81^a^^,^^b^^,^^c^	24.666	<0.001**
Paraplegia	166±105	173±104^b^	126±93	142±104	160±116	215±233	200±206^b^	2.176	0.187
Tetraplegia	127±100	132±109^a^	107±120	137±169	97±52	108±54^a^	128±122^a^	0.438	0.851
F value (ANOVA)	0.488	9.537	2.211	0.045	2.577	4.994	10.916		
p value	0.618	0.001*	0.098	0.956	0.091	0.013*	<0.001*		

* Statistical significance of ANOVA analysis.

** Statistical significance of RM-ANOVA analysis

^a^ p<0.05, ANOVA test with post hoc test by Tukey’s method, values at p2, p6 and p7 were significantly different between normal and tetraplegia groups (statistical powers are 0.91, 0.99, and 0.99)

^b^ p<0.05, ANOVA test with post hoc test by Tukey’s method, values at p2 and p7 were significantly different between normal and paraplegia groups (statistical powers are 0.72 and 0.69)

^c^ p<0.05, repeat measurement ANOVA test, values of p2, p6 and p7 were significantly different from baseline (p1) in normal group (statistical powers are 0.95, 0.99, 0.99)

We found that there were some tetraplegic subjects with more prominent SBP elevations due to severe AD reaction which aroused queries that if these patients would have more prominent HRV changes. Then we divided the tetraplegic subjects into two subgroups according to whether their SBP elevation was larger than 50 mmHg during UDS. Five subjects presented SBP elevation larger than 50 mmHg, while the other seven subjects had no such presentation. Subjects with significant SBP elevation (>50 mmHg) demonstrated with significant elevated LF/HF ratio (p6: 146±72, p7: 249±131 versus p1: 81±39, p<0.05) near maximum bladder capacity (p6 and p7) which is similar to the responses of able-bodied subjects.

Only one tetraplegia patient suffered from febrile reaction compatible with post UDS UTI after laboratory test. The UTI episode subsided after oral antibiotic treatment for one week. The incidence rate of post UDS UTI is 1/36 (2.78% in all study participants) and 1/24 (4.17% in SCI patients).

## Discussion

We acknowledge that this was the first report to use HRV as a tool to evaluate and compare autonomic reactions during UDS in SCI patients and able-bodied controls. Increased LF/HF ratio, which indicates the sympathovagal balance shifting to a sympathetic component, is clearly noted as able-bodied controls approach maximum bladder capacity. On the contrary, SCI patients, including both paraplegic and tetraplegic groups, showed no significant changes in their HRV (LF/HF ratio) as compared to baseline. Tetraplegic SCI patients had marked increase in BP and PR during bladder distension, but only patients with AD and SBP elevation >50 mmHg demonstrated similar LF/HF responses as the able-bodied controls. It is proposed that profound AD may facilitate sympathetic activation controlling heart rate, which is presented in changes of HRV.

When reviewing the literature, Mehnert was the only study to report HRV changes during bladder distension in healthy volunteers[16]. The study reported an abrupt LF/HF elevation at the presentation of strong desire to void (SDV), which we also observed in our able-bodied subjects. Mehnert proposed that a sympathetic tone rise near the SDV point might result from the mental awareness of stress or the vesico-sympathetic reflex. However, Mehnert did not provide the evidence of BP changes during bladder stimulation to verify the study’s hypothesis[16]. In our study, we compared the HRV reactions of able-bodied subjects and SCI patients and found that LF/HF elevated as bladder filling approach fullness seen in the able-bodied group, as in Mehnert’s report, but not in the SCI groups. The tetraplegic group demonstrated elevated BP during bladder distention compatible with an AD event but no definite HRV changes. Thus we attempted to further explore and explain the underlying mechanism of HRV and its relationship with AD reactions in SCI patients.

Bladder distension is perceived in able-bodied subjects through small diameter mechanoreceptive fibers (mainly Aδ fibers). The impulse travels to lamina I neurons of the dorsal horn, which project to (1) the sympathetic cell column of the thoracolumbar spinal cord, containing sympathetic preganglionic neurons, and (2) to higher control centers. These higher control centers include brainstem homeostatic integration sites, the parabrachial nucleus, and the paraqueductal gray, which also receive parasympathetic afferents are interconnected with the hypothalamus and the amygdale complex[22]. Supraspinal activation of T10-L2 sympathetic bladder motor neurons and inhibition of S2-S4 parasympathetic bladder motor neurons are essential during the storage phase of able-bodied subjects. Thus, supraspinal bladder sympathetic neuron activation during bladder storage may at least partially contribute to sympathetic predominance presenting with the phenomenon of elevated LF/HF toward p7 in able-bodied subjects. Furthermore, HRV changes to bladder stimulation might be a normal neurological response involving the higher neurological center, which is disrupted in complete SCI patients. In this study, we also found that the elevated LF/HF spike demonstrated during Foley catheter insertion in able-bodied subjects is not shown in SCI groups. This phenomenon may be caused by uncomfortable catheterization procedure, which usually is not perceived due to the lack of bladder sensation in SCI groups. In our experience, our SCI patients had more frequent AD reactions happened to bladder fullness than simple Foley catheter insertion, which they do several times daily for bladder emptying via intermittent catheterization. We speculate that the discomfort or the nerve impulse from perception of Foley catheter insertion may be much less than bladder fullness. However in able-bodied controls, the perception of Foley catheter insertion is stronger or similar to bladder fullness.

Compared with able-bodied subjects, our tetraplegic patients had markedly elevated SBP with increasing bladder capacity compatible with AD reaction, which can be explained by the vesico-sympathetic reflex. Although the SBP near SDV of our able-bodied subjects was not elevated as much as the tetraplegic group, the SBP elevation still remained statistically significant (p<0.05). According to our results, able-bodied subjects presented with LF/HF elevation and marginally statistical SBP increase. Vesico-sympathetic reflex could only partially contribute to able-bodied autonomic response in HRV during UDS. Vesico-sympathetic reflex is a kind of viscero-sympathetic reaction was first addressed by Sherrington in 1899, who mentioned that stretching of certain hollow viscera, ureter, bile duct, and urinary bladder causes reflex vascular responses and an elevation of BP[23]. Subsequent research has also found that bladder filling could induce reflex increase in arterial pressure and peripheral vascular resistance[23–25]. These reactions may augment outflow in sympathetic nerves and were demonstrated in both able-bodied and SCI subjects[23–26]. It is plausible that C-fiber bladder afferents, which usually do not respond to bladder filling phase, become mechano-sensitive and initiate automatic micturition after SCI[27]. When the SCI patients with a lesion above T6, the reflex will be exaggerated; this is known as AD[6,28]. In addition, it was reported that intravesical administration of afferent neurotoxins, capsaicin or resiniferatoxin, reduced AD and detrusor hyperreflexia in SCI patients[29,30]. These observations suggest that capsaicin-sensitive C-fiber may be involved in AD. Zahner had also provided strong evidence to support the hypothesis that the cardiogenic sympathoexcitatory reflex is mediated by capsaicin-sensitive afferent fibers[31]. Therefore, we infer that bladder filling activates the vesico-sympathetic reflex via capsaicin sensitive nerves to project ascending input to the dorsal horn of the T1-T5 sympathetic cord in tetraplegia patients. This abrupt BP elevation during bladder filling in tetraplegic patients may trigger the baroreceptor to activate parasympathetic reaction via the vagus nerve. The vagal control of the heart originates in the medulla and usually is spared after SCI, and vagal tone at rest is essentially increased after cervical SCI[1]. During AD reactions, exaggeration in both the sympathetic and vagus tone innervations of the heart make the heart rate change more variable and less predictable[6]. The vagus nerve innervates only to the heart, with no effect on blood vessel function[32]. This may explain the discrepancy between BP and HRV changes of the tetraplegic group during the bladder distension. In contrast, paraplegia patients, with disruption of afferent spinal input to T1-T5 sympathetic neurons, revealed stable HRV and BP over the bladder filling. In summary, in addition to the vesico-sympathetic reflex as able-bodied subjects, the reactivation of silent c-fibers via noxious bladder distention or other pathways in tetraplegic SIC patients during AD needs to be considered and further elucidated[33].

Although differences exist between the neurological pathway of AD and HRV, strong AD reaction could still induce HRV changes. The tetraplegic patients with SBP elevation >50 mmHg during bladder filling also experienced increased LF/HF when approaching bladder fullness, a similar to the reaction in able-bodied subjects. Previous studies of SCI patients show that the LF component may partially increase because of augmented sympathetic activities, presumably due to the excitation of the isolated spinal sympathetic nervous system in response to the various stimuli from the periphery below the level of the lesion[34]. AD was found to associate with elevation of circulatory noradrenaline, and studies have documented that this increase in plasma noradrenaline level derives mostly from peripheral sympathetic nerve endings while the plasma adrenaline from the adrenal medulla is usually unchanged[35,36]. Massive circulating noradrenaline may prompt the increase of sympathetic activity of the heart leading to elevated LF/HF at the end of bladder filling.

Some researchers reported that HRV was affected by mean heart rate (HR). Sacha et al. demonstrated that the HF component of HRV was inversely, while the LF component was directly, related to average baseline HR of the subject[37–40]. They considered, even though only 30% HRV could be attributed to HR, HRV data correction was important to remove any mathematical bias from HRV calculations. Billman used pharmacologic and surgical disruptions of cardiac parasympathetic nerve to produce similar increases in HR and reduction of HRV and exercise to provoke increases in HR accompanied by decreases in HRV. However, HRV to these interventions was not altered by correction for prevailing HR[41]. In general, it is possible that for outcomes and populations where HR is not a risk factor, the removal of HR impact improves the HRV predictive value. Conversely if HR is a risk factor, the enhancement of its influence makes HRV a better predictor[42].

SCI patients may present with AD when bladder is filled during UDS. Comparing with previous BP measurement and clinical symptoms observation, we want to know whether HRV could provide reliable and immediate information to detect AD events as soon as possible to minimize the dreadful sequelae in SCI patients during urodynamic study. But the results in our preliminary report are suboptimal. Not all tetraplegic patients with AD, but only SBP elevation > 50 mmHg subjects, could demonstrate LF/HF elevation near maximum bladder capacity. HRV monitoring may be useful in those SCI patients with significant AD during UDS. These SCI patients have the highest risk to develop dreadful disaster of AD during UDS. Whether concomitant BP and HRV recording provide more accurate information in AD detection, it needs further studies to elucidate.

### Study Limitations

Our study experienced some limitations. We classified enrolled SCI patients according to American Spinal Injury Association criteria with single and complete cord lesion. The completeness of lesion was judged by sensory and motor tests rather than a sympathetic test. We did not perform sympathetic skin test to differentiate the possible preserved pathways. Furthermore, the circulatory noradrenaline level was not examined in tetraplegic patients to prove the contribution of this transmitter in sympathetic activity during AD. Small study subjects, particularly only five patients per subgroup, may also limit our analysis with respect to LF/HF elevation in tetraplegia patients with severe AD. Lastly, we did not correct HRV data according to baseline HR. Yet, HR is expected to affect both frequency components equally[43]. Some investigators argue that LF/HF is the best measure of autonomic nerve activity to the heart.[21] Our main outcome parameter in HRV is LF/HF, which is probably not significantly influenced by HR. Furthermore, HRV correction studies only enroll able-bodied subjects, which require that the usefulness of SCI patient to be further elucidated. Consequently, correcting the HRV data does not seem to affect the association in this report.

## Conclusions

Tetraplegic patients experienced BP elevation but did not experience changes in HRV during bladder distension. This finding may imply that different neurological pathways contribute to AD reaction and HRV changes during bladder distension. However, profound AD (SBP increment >50 mmHg) during UDS in tetraplegic patients was associated with corresponding changes in HRV. Whether HRV monitoring would be beneficial in SCI patients presenting with significant AD, it needs further studies to elucidate.
